# *Longidorus carniolensis* sp. n. (Nematoda, Longidoridae) from vineyard soil in Slovenia

**DOI:** 10.3897/zookeys.141.1906

**Published:** 2011-10-28

**Authors:** Saša Širca, Gregor Urek, Stela Lazarova, Milka Elshishka, Vlada Peneva

**Affiliations:** 1 Agricultural Institute of Slovenia, Hacquetova ulica 17, 1001 Ljubljana, Slovenia; 2 Institute of Biodiversity and Ecosystem Research, Bulgarian Academy of Sciences, 2, Yu. Gagarin Street, 1113 Sofia, Bulgaria

**Keywords:** grapevine, morphology, taxonomy, 28S rDNA

## Abstract

A new needle nematode, *Longidorus carniolensis* **sp. n.**, recovered from the soil around the roots of grapevine *Vitis vinifera* L. from Slovenia, is described and illustrated. *Longidorus carniolensis*is an amphimictic species**,** characterised by females with a moderately long (L=5.6–8.2 mm) and plump (a=51–72.4, ave. 66.3) body, assuming a spiral to C-shape when heat relaxed. Head region continuous, anteriorly almost flat, lip region 23–25 µm wide; guiding ring situated posteriorly (42–47 μm, 43–50 μm in males), odontostyle long (ave. 146.6 (136–157) μm); pharyngeal glands with normal location, their nuclei of approximately equal size; tail bluntly conoidal to almost hemispherical. Males abundant, spicules slender and long (122–145 μm), ventromedian supplements 13–17, irregularly spaced, preceded by an adanal pair. Four juvenile stages present, the first stage juvenile with bluntly conoidal tail. Codes for identifying the new species when using the key by Chen et al. (1997) are: A 56, B 4, C 4, D 1, E 4, F 35, G 1, H 1, I 2. The new species is morphologically the most similar to *Longidorus poessneckensis* Altherr, 1974, *Longidorus macrosoma* Hooper, 1961, *Longidorus caespiticola* Hooper, 1961, *Longidorus helveticus* Lamberti et al., 2001, *Longidorus macroteromucronatus* Altherr, 1974, *Longidorus pius* Barsi & Lamberti, 2001, *Longidorus raskii* Lamberti & Agostinelli, 1993, *Longidorus kheirii* Pedram et al. 2008, *Longidorus silvae* Roca, 1993, *Longidorus iuglandis* Roca et al., 1985, *Longidorus vinearum* Bravo & Roca, 1995 and *Longidorus major* Roca & d’Erico, 1987, but differs from these species either by the body and odontostyle length, position of guide ring, head region and tail shape or the shape of the first stage juvenile tail. Sequence data from the D2-D3 region of the 28S rDNA distinguishes this new species from other speciesof the genus *Longidorus* with known sequences. Relationships of *Longidorus carniolensis* **sp. n.** with other *Longidorus* species based on analysis of this DNA fragment and morphology are discussed.

## Introduction

The nematodes of the genus *Longidorus* Micoletzky, 1922 cause damage to many economically important crops by direct feeding on their roots. Additionally, they can cause indirect damage to the host plants by transmitting plant viruses. To date, six *Longidorus* species have been reported from Slovenia (Širca and Urek 2009): *Longidorus elongatus* (de Man, 1876) Micoletzky, 1922, *Longidorus caespiticola* Hooper, 1961, *Longidorus juvenilis* Dalmasso, 1969, *Longidorus helveticus* Lamberti, Kunz, Grunder, Molinari, De Luca, Agostinelli & Radicci, 2001, *Longidorus leptocephalus* Hooper, 1961, *Longidorus moesicus* Lamberti, Choleva & Agostinelli, 1983. The study of relationships between longidorids and Nepoviruses in Slovenia and Bulgaria in the frame of a bilateral project, revealed the presence of a new species described herein as *Longidorus carniolensis* sp. n. The description of the new species is based both on morphological and molecular data, in particular the sequence of D2D3 expansion regions of the large subunit rDNA nuclear gene which proved to be useful in molecular phylogenetic analyses of Longidoridae (Rubtsova et al. 2001, Ye et al. 2004, He et al. 2005). Additionally, sequences of these domains allow species differentiation (Širca and Urek 2009).

## Materials and methods

Soil samples were collected in July 2008 and October 2009 from the rhizosphere of *Vitis vinifera* L. in Drašiči and Krmačina localities in the southern part of Slovenia. The sampling was performed by digging holes beneath grapevine plants and carefully collecting soil around the roots at 40–50 cm depth. Approximately 500 cm^3^ of a collected soil sample was gently mixed and two 200 cm^3^ sub-samples were processed. Nematodes were extracted from the soil using a decanting method followed by the Baermann funnel technique. Longidorid nematodes for morphological study were hand-picked, fixed in TAF (7 ml 40% formalin, 2 ml tri- ethanolamine, and 91 ml distilled water), processed to glycerol (Seinhorst 1959) and mounted on glass microscope slides in anhydrous glycerol.

Drawings and photographs were taken using an Olympus BX51 compound microscope powered with differential interference contrast (DIC). Images were taken with a ColorView IIIu camera and cell^P software (Olympus Soft Imaging Solutions Gmbh). Measurements were made using an Olympus BX 41 light microscope, a digitising tablet (CalComp Drawing Board III, GTCO CalCom Peripherals, Scottsdale, AZ, USA), and Digitrak 1.0f programme (Philip Smith, Scottish Crop Research Institute, Dundee, UK).

### Total DNA extraction and amplification

Extracted female nematodes for molecular study were transferred into 1.5 ml tube in a 1 μl drop of sterile water. DNA was extracted from a single female nematode from type-locality Drašiči and from Krmačina locality; 10 ml 1M EDTA pH 8 and 50 ml nucleic lysis solution (Promega Wizard DNA purification kit) mixture was added to each tube and homogenised with micropestle. Isolation of DNA was continued according to manufacturer’s instructions. Isolated DNA was re-suspended in 10 µl of distilled water of which 2 µl was used in each PCR reaction. A fragment of the D2 and D3 expansion region of the 28S rDNA gene was amplified using the primers D2A (5’-ACA AGT ACC GTG AGG GAA AGT TG-3‘) and D3B (5‘-TCG GAA GGA ACC AGC TAC TA-3‘) (Rubtsova et al. 2001) in a PCR cycler and conditions as described earlier (Širca et al. 2007).

### Analyses of rDNA sequence

Obtained PCR products were purified using the JetQuick PCR purification spin kit (Genomed) and sequenced on an ABI PRISM 310 DNA Sequencer using BigDye Terminator Cycle Sequencing Ready Reaction Kit (Applied Biosystems), the sequences obtained were submitted to the GenBank. Cluster analyses were performed using sequences of several *Longidorus* species from the NCBI GenBank (http://www.ncbi.nlm.nih.gov/) obtained from different phylogenetic studies (Rubtsova et al. 2001, Handoo et al. 2005, He et al. 2005, Lišková 2007, Kumari et al. 2009, Širca and Urek 2009) (Table 1). *Xiphinema index* (AY601628) (He et al. 2005) was used as an out-group. For cluster analyses and tree construction a Neighbour-Joining method was applied using MEGA5 software (Tamura et al. 2011).

**Table 1.** Species of fam. Longidoridae used in phylogenetic reconstructions.

**GenBank accession number**	**Nematode species**	**Origin**	**Reference**
AY601583	*Longidorus africanus* Merny, 1966	California, USA	He et al. 2005
AY494715	*Longidorus americanum* Handoo, Carta & Skantar, 2005	Georgia, USA	Handoo et al. 2005
AY601571	*Longidorus apulus* Lamberti & Bleve-Zacheo, 1977	Mola di Bari, Italy	He et al. 2005
AY601570	*Longidorus arthensis* Brown, Grunder, Hooper, Klingler & Kunz, 1974	Suter, Switzerland	He et al. 2005
AY601574	*Longidorus athesinus* Lamberti, Coiro & Agostinelli, 1991	Italy	He et al. 2005
AY601572	*Longidorus attenuatus* Hooper, 1961	Germany	He et al. 2005
AY601576	*Longidorus breviannulatus* Norton & Hoffmann, 1975	Nebraska, USA	He et al. 2005
HM447030	*Longidorus caespiticola*	Brdo, Slovenia	Širca and Urek 2009
AY601585	*Longidorus camelliae* Zheng, Peneva & Brown, 2000	Hangzhou, China	He et al. 2005
JN631811	*Longidorus carniolensis* sp. n.	Krmačina, Slovenia	This study
JN631812	*Longidorus carniolensis* sp. n.	Drašiči, Slovenia	This study
AF480072	*Longidorus carpathicus* Lišková, Robbins & Brown, 1997	Germany	Rubtsova 2001
EF654539	*Longidorus distinctus* Lamberti, Choleva & Agostinelli, 1983	Kráľovský Chlmec, Slovakia	Lišková 2007
AY593057	*Longidorus dunensis* Brinkman, Loof & Barbez, 1987		Holterman et al. Unpublished
AY601575	*Longidorus edmundsi* Hunt & Siddiqi	Caribbean sea beach, Cuba	He et al. 2005
HM447032	*Longidorus elongatus*	Maribor, Slovenia	Širca and Urek 2009
AY601573	*Longidorus euonymus* Mali & Hooper, 1973	Zabagr, Hungary	He et al. 2005
AY601581	*Longidorus goodeyi* Hooper, 1961	Peebles, Scotland, UK	He et al. 2005
HM447031	*Longidorus helveticus*	Trška gora, Slovenia	Širca and Urek 2009
AF480074	*Longidorus intermedius* Kozlowska & Seinhorst, 1979	Germany	Rubtsova et al. 2001
DQ364599	*Longidorus juvenilis*	Svetinje, Slovenia	Širca et al. 2007
AY601568	*Longidorus latocephalus* Lamberti, Choleva & Agostinelli, 1983	Greece	He et al. 2005
DQ364600	*Longidorus leptocephalus*	Juršinci, Slovenia	Širca et al. 2007
AY601565	*Longidorus macrosoma* Hooper, 1961	Switzerland	He et al. 2005
HM447029	*Longidorus moesicus*	Vrhpolje, Slovenia	Širca and Urek 2009
AY601577	*Longidorus piceicola* Lišková, Robbins & Brown, 1997	Branisko, Slovakia	He et al. 2005
EF538750	*Longidorus poessneckensis* Altherr, 1974	Cerne Voderady, Czech Republic	Kumari et al. 2009
AF480073	*Longidorus profundorum* Hooper, 1965	Germany	Rubtsova et al. 2001
AF480071	*Longidorus sturhani* Rubtsova, Subbotin, Brown & Moens, 2001	Belgium	Rubtsova et al. 2001
EF538754	*Longidorus uroshis* Krnjaic, Lamberti, Krnjaic, Agostinelli & Radicci, 2002	Velke Pole, Slovakia	Kumari et al. 2009
AY601628	*Xiphinema index* Thorne & Allen	Argentina	He et al. 2005

## Taxonomy

### Longidorus carniolensis sp. n.

urn:lsid:zoobank.org:act:546D321E-CF14-46C1-A623-73A1F76839BD

http://species-id.net/wiki/Longidorus_carniolensis

Figs 1 [Fig F2] [Fig F3] [Fig F4] [Fig F5] [Fig F6] [Fig F7] [Fig F8] [Fig F9] [Fig F10] [Fig F11] 12

#### Measurements.

See Table 2.

**Table 2.** Measurements of females, males and juvenile stages of *Longidorus carniolensis* sp. n., from Slovenia (mean ± standard deviation, with range). All measurements in micrometers.

**Character**	**Holotype**	**Females**	**Males**	**J1**	**J2**	**J3**	**J4**
n	n=1	n=13	n=14	n=15	n=6	n=11	n=9
L	7089	7447.5±679.0 5653-8226	7917.7±753.9 6702-9525	1349.9±53.8 1283-1449	2584.2±228.0 2329-2872	3692.2±238.0 3305-4149	5441.2±700.7 4677-6647
a	65.2	66.3±6.1 51.0-72.4	72.5±6.4 59.6-81.2	44.0±2.3 39.3-46.2	46.5±4.3 39.8-50.6	50.5±4.7 42.8-57.4	60.6±3.7 53.2-65.6
b	14.2	12.7±0.9 11.6-14.3	12.8±0.8 11.8-14.9	4.9±0.4 4.3-5.6	6.3±0.3 5.8-6.6	7.9±0.7 7.0-9.0	9.7±1.7 8.4-13.9
c	165.7	177.9±35.5 108.1-224.5	173.7±27.5 127.6-241.8	41.2±2.7 36.8-45.9	71.9±4.8 66.4-79.4	94.2±9.2 82.5-113.6	132.6±17.6 98.2-155.1
c’	0.6	0.6±0.1 0.5-1.0	0.8±0.1 0.6-1.1	1.4±0.1 1.2-1.5	0.8±0.04 0.7-0.9	0.7±0.1 0.6-0.8	0.7±0.04 0.6-0.8
V (%)	49.7	49.4±1.4 47.1-51.5					
G1 (%)	11.9	13.4±2.6 10.6-17.4					
G2 (%)	11.8	13.3±2.6 9.9-17.3					
d	2.0	1.8±0.1 1.7-2.0	2.1±1.1 1.0-5.8	2.0±0.1 1.8-2.2	1.9±0.1 1.8-2.1	1.9±0.1 1.7-2.1	1.7±0.6 0.3-2.0
d’	2.0	2.0±0.2 1.8-2.3	1.9±0.1 1.8-2.1	1.7±0.1 1.6-1.9	1.8±0.2 1.7-2.1	1.9±0.1 1.7-2.2	1.9±0.4 0.8-2.2
Anterior end to guiding ring	45.4	44.6±1.6 42-47	46.5±2.3 43-50	21±0.7 20–22	28.7±1.6 26.5-30.5	33.9±1.0 32-35	39.8±1.2 38-42
Anterior end to nerve ring	267.5	258.6±15.4 220-275	270.7±9.5 249-289	129.6±8.9 114-145	182.1±31.0 153-228	207.5±24.1 176-273	231.1±15.9 213-252
Hemizonid	237.5	253.7±18.9 204-270	10.4±0.5 10-11			205.9±7.4 195-216, n=7	234.6±19.2 210-216, n=4
Odontostyle	144	147.5±4.7 136-157	149.1±6.6 132-159	81.9±3.5 76-88	88.2±5.0 79-94	107.7±4.4 98-114	125.9±3.6 120-131
Replacement odontostyle				87.6±2.5 85-93	104.9±2.4 101-108	123.7±3.2 119-130	146.0±3.9 142-152
Odontophore	92	91.4±4.1 85-97	90.2±6.4 75-99	49.6±3.9 43-56	62.4±2.3 59-66	72.9±4.2 64-78	85.1±3.4 81-91
Neck length	500	590.5±59.9 462-674	618.6±56.6 510-703	274.1±20.8 237-317	408.3±33.4 370-448	472.5±48.7 409-524	553.6±47.5 478-628
Pharyngeal bulb length	139	142.7±6.0 133-152	141.1±6.9 129-157	72.6±2.3 67-75	89.6±3.1 84-93	106.8±6.1 96-114	123.6±5.6 118-135
Pharyngeal bulb width	39	40.2±2.8 36-44	37.9±2.1 32-41	15.6±0.9 14-17	22.7±1.4 21-24	28.1±1.6 26-31	33.3±1.9 31-37
DO*	11.4	11.6±0.3 11.2-11.9	11.0±0.8 9.8-11.8	14.0±2.4 11.3-18.1	10.9±1.3 9.1-12.0	11.3±0.7 10.3-11.9	11.7±1.6 9.9-14.8
DN	37.1	38.1±2.6 32.8-40.2	37.9±3.0 33.2-42.9	38.6±1.9 36.7-42.7	37.2±1.7 35.5-40.3	37.0±1.0 35.6-38.4	36.8±3.7 30.0-42.6
LS1N	53.7	56.1±2.6 52.3-61.0	55.0±3.0 49.6-62.3	52.0±2.6 47.9-57.3	50.6±1.7 48.6-52.7	52.9±2.1 48.1-55.3	54.3±2.2 52.2-57.7
RS1N	53.7	54.6±2.5 51.5-58.6	55.5±2.6 52.4-61.2	51.5±2.7 46.3-55.3	50.9±1.4 48.9-52.7	53.3±2.3 48.1-56.3	53.5±2.5 50.7-57.4
S2O	83.4	84.9±1.6 82.3-87.1	86.3±5.7 81.9-102.5	84.2±1.0 82.7-85.8	84.8±2.7 83.1-90.2	85.0±1.6 81.6-87.1	82.8±0.9 81.6-84.1
Prerectum	347.5	419.0±85.9 280-550	576.8±182.2 248-832	141.7±60.7 81-290	183.8±30.1 150-224	289.1±50.0 175-363	363.6±91.7 275-538
Rectum	51.5	50.3±3.0 47-57	-	14.3±1.9 12-19	22.8±0.8 22-24	32.6±2.6 30-38	45.3±2.4 42- 49
Tail	42.8	43.3±9.2 34-69	46.4 ± 6.8 32-61	32.9±1.4 31-35	35.9±1.6 34-38	39.4±2.9 35.5-45	41.2±3.2 38-48
Length of hyaline part	20.2	17.8±0.7 17-19	16.9±1.2 14-19	7.9±0.9 6-9	10.4±0.7 9-11	13.1±0.9 11-15	14.9±0.8 13-16
Body diameter at: - lip region	22.9	24.2±0.8 23-25	24.8±1.3 22-26.5	10.6±0.4 10-11	15.0±0.5 15-16	18.4±1.1 17-20	20.9±0.5 20-21
- guiding ring	46.6	48.5±3.3 44-55	48.0±2.3 45-52	18.3±0.5 18-19	26.8±2.2 25-31	35.7±2.7 31-41	43.0± 3.9 38.7-51
- base of pharynx	95.4	93.4±4.5 89-101	93.8±4.8 84-103	30.2±1.2 29-33	50.5±3.4 46-55	63.9±3.8 59-73	76.4±5.6 69.5-84
- mid-body/at vulva	108.7	112.9±9.5 97-127	109.3±5.4 98-117	30.7±1.2 29-33	55.8±4.0 49-60	73.5±5.6 66-85	89.8±10.0 79-105
- anus	68.5	68.2±4.3 60-75	61.2±3.2 55-66	24.3±1.0 22-26	44.4±1.7 42-46	55.2±3.6 51.3-60.5	63.1±3.6 59-70
- hyaline part	49.5	49.0±1.7 47-52	39.5±2.2 37-43	16.0±0.9 15-17	26.8±2.7 24-32	37.6±2.5 32-41	42.9±2.7 39-47
Spicules			126.9±5.8 122-145				

* Following Loof and Coomans 1972

#### Description.

**Figure 1.**
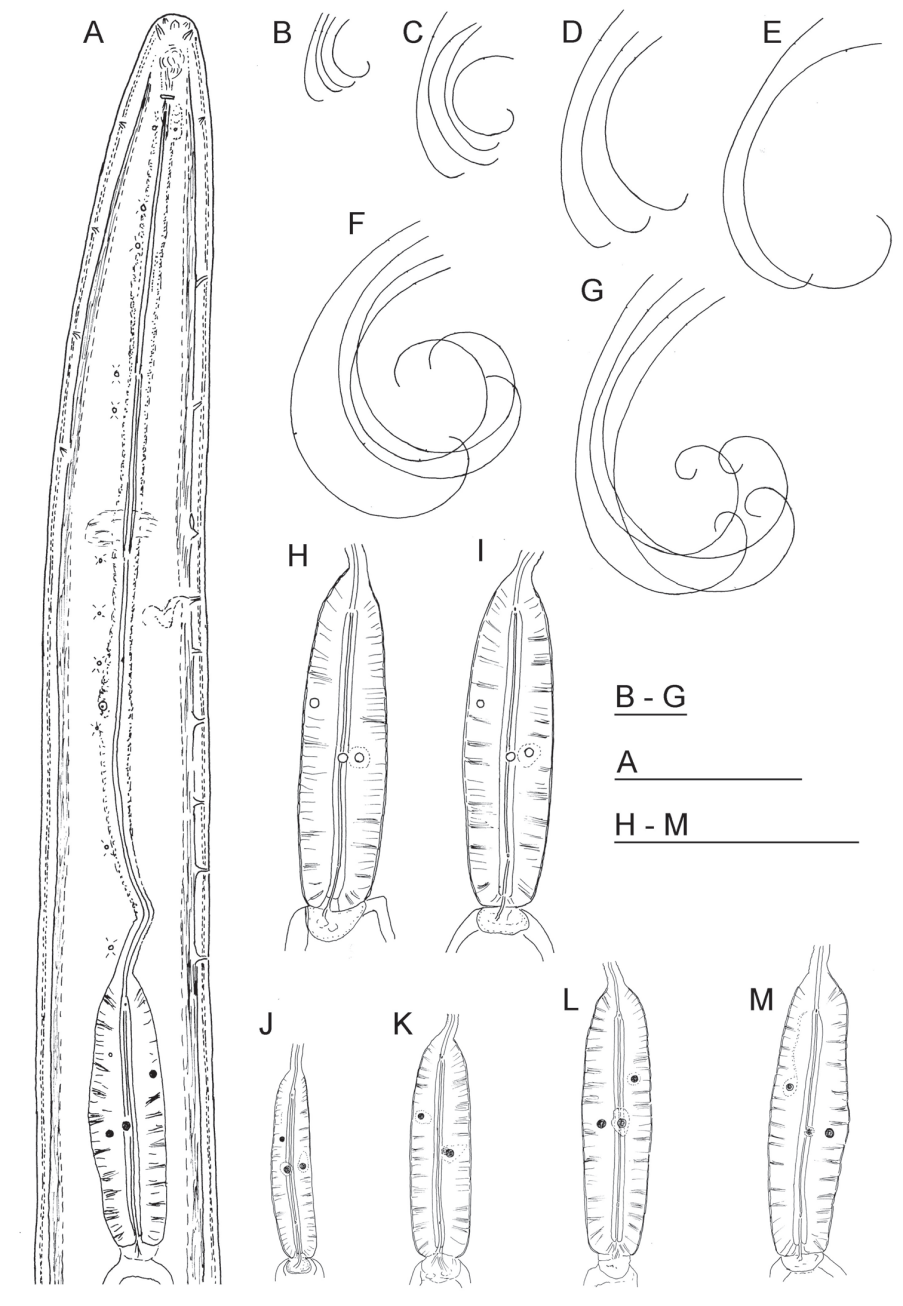
*Longidorus carniolensis* sp. n. *Female*: **A** Neck region **F** Habitus **H** Pharyngeal bulb *Male*: **G** Habitus **I** Pharyngeal bulb; *Juveniles*: **B–E** Habitus of first, second, third and forth juvenile stages **J–M** Pharyngeal bulb of first, second, third and forth juvenile stages. Scale bars: **B–G** 1 mm; **A, H–M** 100 μm.

**Figure 2. F2:**
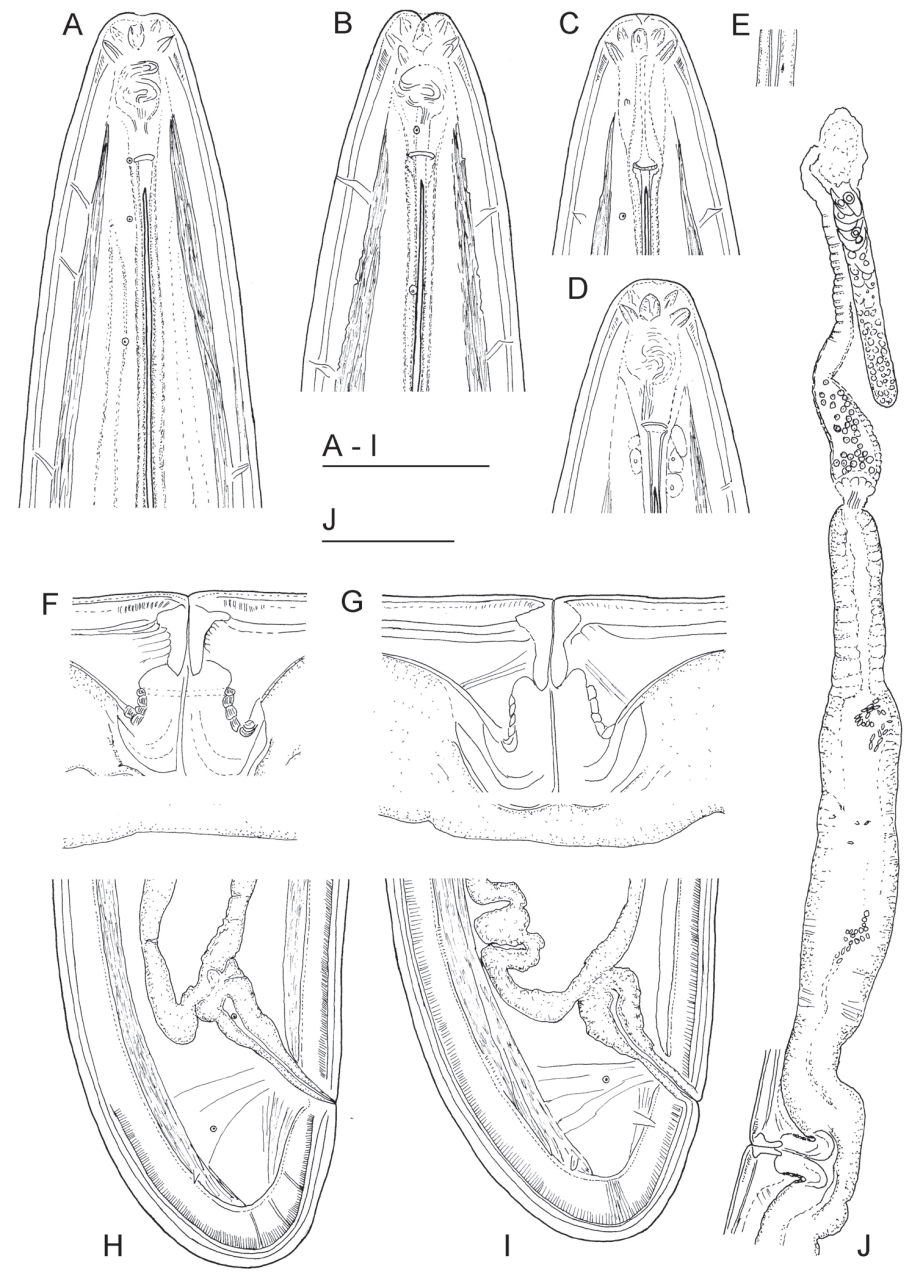
*Longidorus carniolensis* sp. n. *Female*: **A–D** Anterior ends **E** Vestigium in the walls of the slender part of pharynx **F, G** Vulval region **G** Anterior genital branch. *Scale bars*: **A–I** 50 μm, **J** 100 μm.

*Female*. Body moderately long (L=5.6–8.2 mm) and plump (a=51–72.4), assuming a spiral to C shape when heat relaxed. Cuticle consisting of several layers under light microscope: 11–14 µm thick at guiding ring level; 7–8 µm along the body; 13–15 µm on tail posterior to anus. Lateral pores number 10–14 in pharyngeal region: a single pore in front of guide ring, rarely two or none; 3–5 in odontostyle and 1–3 in odontophore regions; 3–4 dorsal pores and 7–10 ventral pores; numerous lateral body pores. Usually the fifth ventral pore (sometimes the fourth) differs in size (Figs 1A, 4F and 6H) compared to the other ventral pores. Lip region continuous, anteriorly almost flat, 7–9 μm high. Labial papillae prominent. Amphid aperture assumed to be a minute pore, difficult to be observed under light microscope. Pouch-like amphidial fovea with convoluted fine dendritic branches (receptors), extending to 1/2 - 2/3 the distance between anterior end and guiding ring, fovea slightly longer (15–18 μm, n=5) than wide (14–16 μm, n=4) with no distinct margins. Fusus (sensillium pouch) at 57±1.9 (55–60) μm from anterior end. Guiding ring 7–9 μm wide. Odontostyle long and very slender, 2 μm wide at the base. Odontophore with weakly developed flanges. In all females a small (2–3 μm long) rudimentary odontostyle tip (vestigium) present, directed forward, and observed in the slender pharynx at 300.5±40.3 (224–350) μm from anterior end; in two specimens the vestigium located in odontophore area. Slender pharynx often coiled in its posterior part. In this region 5–7 glandular bodies are observed in all females. Nerve ring surrounding odontophore base, rarely surrounding mid-odontophore, or just behind it, second nerve ring at a distance of 85.2±6.6 (78–98) μm behind the first one. Hemizonid flat, 10–11 μm long. Pharyngeal bulb about 1/4 of the neck length. Normal arrangement of pharyngeal glands, the nuclei of dorsal and ventrosublateral glands approximately the same size, their diameters 3.4±0.4 (3–4) μm, n=7 and 3.9±0.2 (3.5–4) μm, n=11, respectively. Cardia small, broadly rounded, wider than long, variable in size: 20.1±1.8 (10–23) × 10.1±1.8 (7–12) μm . Reproductive system amphidelphic, varying in dimensions due to the stage of maturity of female. Vagina extending about half body width. *Pars distalis vaginae* with characteristic shape (Fig. 2F, G), 26–28 µm and *pars proximalis vaginae* 32–38 µm long, respectively; muscular walls of the latter almost parallel. Uteri very long, anterior uterus 494.6±52 (430–563) µm long, posterior uterus 510.0±88.7 (357–643) µm long, differentiated, filled with sperm cells in all females examined; well developed sphincter between uterus and *pars dilatata oviductus* also containing numerous sperm cells. Anterior and posterior oviduct of similar size, measured in four specimens: 275–348 μm, and 283–330 μm. Anterior ovarium 263.4±51.8 (210–347) μm long, n=7, posterior ovarium 234.3±35.8 (183–309) μm long, n=5; in older mature specimens the length is about 3 times greater (1055–1060 μm for anterior and 1020 μm for posterior ovary). One egg in anterior *pars dilatata oviductus* measuring 227 × 87.5 μm and one uterine egg measuring 225 × 77.5 μm. A weakly developed ovijector present, 112.0±12 (95–125) μm long. In one female a rudimentary adanal pair of supplementary papillae was observed (Fig. 9E). Prerectum variable in length; rectum 0.7±0.1 (0.6–0.8) body width at anus. A short post-intestinal sac present. Tail bluntly conoidal, rounded to almost hemispherical; ventral side straight or slightly convex, the dorsal curvature greater. Two pairs of lateral pores.

*Male*. Body C shaped when heat relaxed, posterior part more strongly coiled ventrally. Similar to females in general morphology except for genital system. Lateral pores number 10–15 in pharyngeal region: a single pore in front of guide ring, 3–5 in odontostyle and 1–2 in odontophore regions; 2–5 dorsal pores, mostly 3–4, and 7–10 ventral pores. Cuticle in post-labial region at the guiding ring level 10.5–13.5 µm thick, 6.5–9 µm along body, 9–10 µm in post-cloacal area. Second nerve ring at 80.7±14 (50–100) μm behind the first one (n=14). In all males a small vestigium (2–3 μm, in one specimen 6 μm long), directed forward (in two specimens directed rearward), is observed in the slender pharynx at 300.5±40.3 (224–350) μm from anterior end; in two specimens the vestigium detected in odontophore area. Two to eleven glandular bodies observed in all males in posterior part of the slender pharynx and pharyngeal bulb. In two specimens lens-like hemizonion at a distance of 242 and 271 μm from anterior end observed. Pharyngeal bulb slightly less than 1/4 of neck length (22.9±1.6 (20.9–26.7%). Ventromedian supplements composed of one adanal pair and a row of 13–17 irregularly spaced single ones, the first three appear as double in some specimens. Spicules comparatively slender, of almost equal width along the length, curved to almost at right angle. Lateral guiding piece not bifid, with uneven internal walls. Post-cloacal papilla well developed. Tail short, bluntly conoidal, ventral side almost straight, dorsal side convex. Two or three pairs of lateral caudal pores.

*Juveniles*. Four developmental stages clearly present (Fig. 11) as determined from the position of the replacement odontostyle and the principal morphometric characters of body, odontostyle and replacement odontostyle lengths, and developing gonad (genital primordium) size. The *habitus* of juveniles not changing considerably during successive stages, assuming J or C shape. In first stage juvenile, lip region somewhat different from the next stages, it is rounded with a very weak depression after the second circle of labial papillae, the latter slightly protruding and changing the lip region outline. Amphidial fovea in first two stages has no clearly visible receptors, only small refractive elements discernable. Both the tail and body width at anus is increasing in length and **c’** ratio is decreasing. Tail shape in J1 is conoidal, ventrally almost straight or slightly concave, dorsally convex, which gives asymmetrical appearance, in successive stages it gradually becomes rounded but always with the dorsal curvature more strongly expressed.

**Figure 3. F3:**
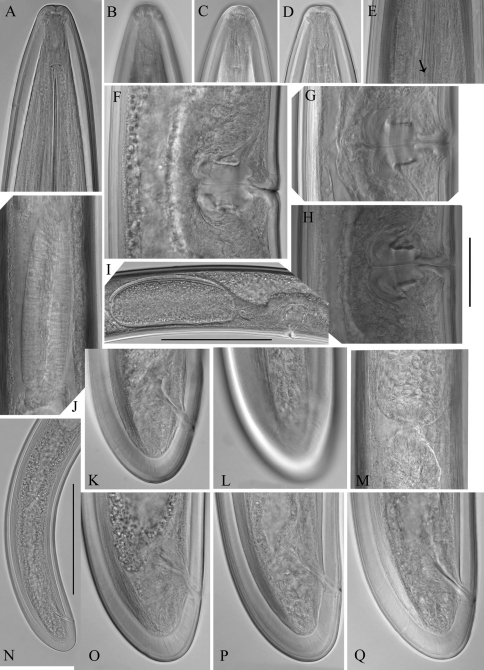
*Longidorus carniolensis* sp. n. *Female:* **A** Anterior region **B–D** Amphidial fovea **E** Vestigium **F–H** Vulval region **I** Vulval region, uterus and egg **J** Pharyngeal bulb, dorsal and subventral glands **K, L** Tail – different optical sections **M** Sphincter **N** Prerectum **O–Q** Variation in tail shape. Scale bars: **I, N** 200 μm; **A–G, H–M,** **O–Q** 50 μm.

**Figure 4. F4:**
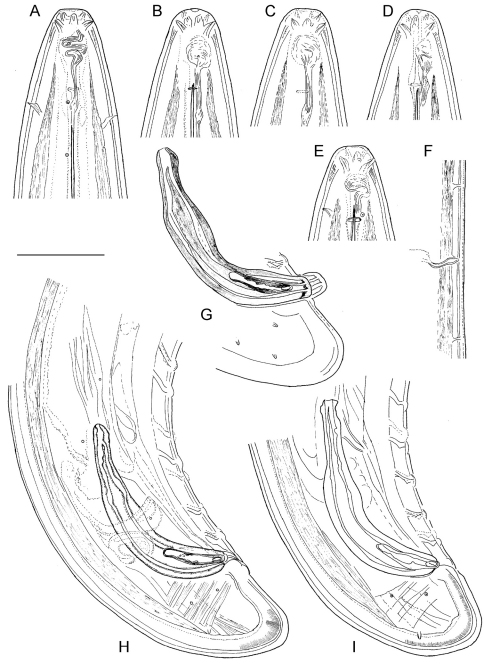
*Longidorus carniolensis* sp. n. *Male:* **A–E** Anterior end **B, D, E** in sublateral view **F** Excretory pore and ventral pores **G** Partly protracted spicules **H–I** Tail end. *Scale bar*: 50 μm.

**Figure 5. F5:**
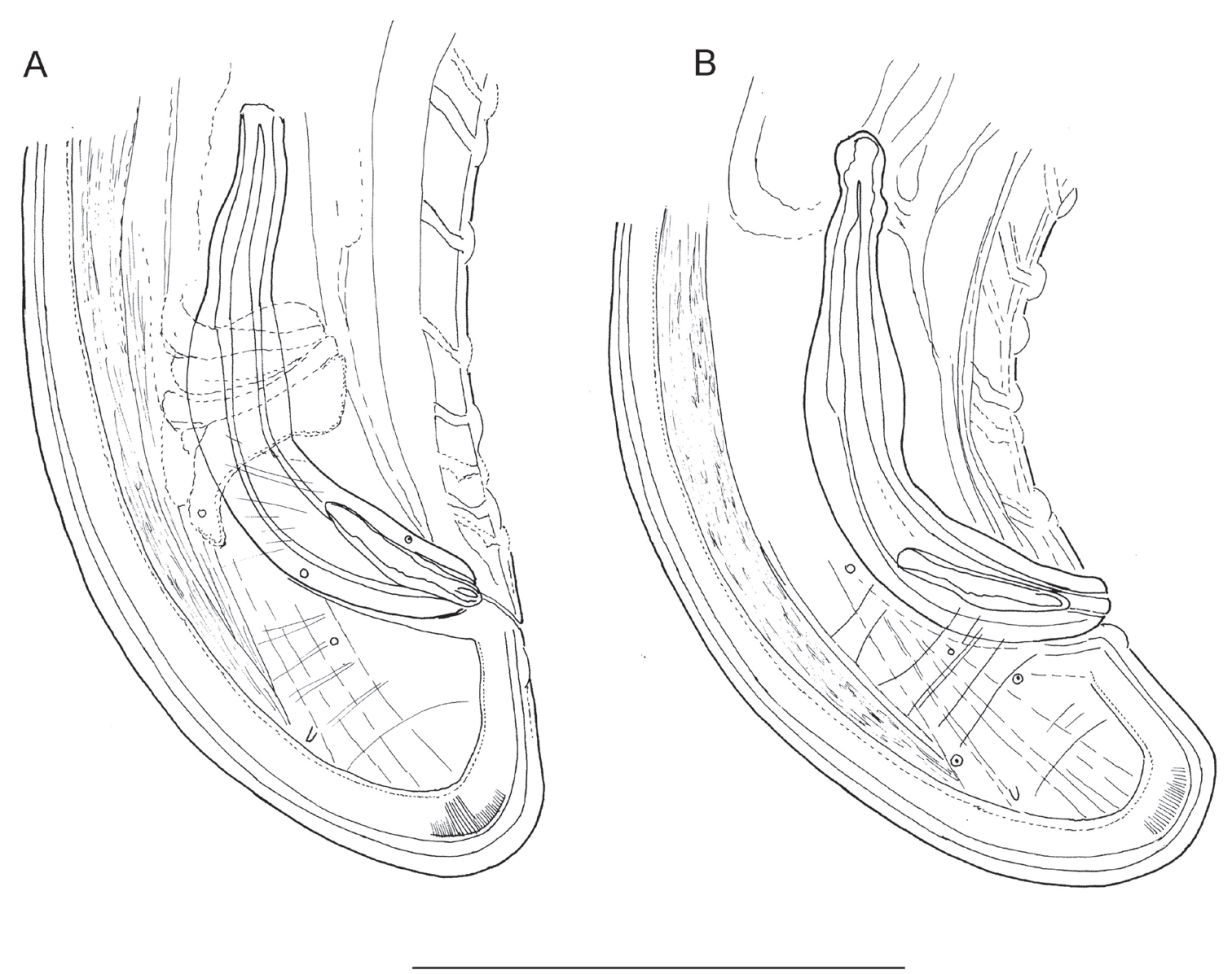
*Longidorus carniolensis* sp. n. Male: **A, B** Variation in tail shape. Scale bar: 50 μm.

**Figure 6. F6:**
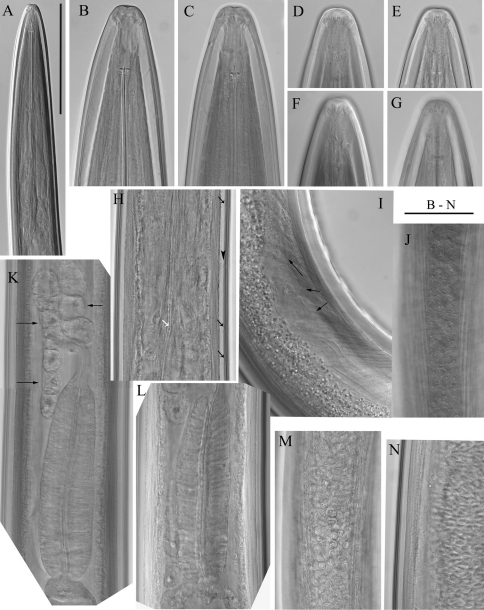
*Longidorus carniolensis* sp. n. *Male:* **A** Anterior region **B, C** Head region **D–G** Amphidial fovea **H** Vestigium (white arrow), excretory pore (thick arrow) and ventral pores (slender arrows) **I** Ejaculatory glands (marked by arrows) **J** Lateral field **K, L** Pharyngeal bulb with glandular bodies (marked by arrows) **M, N** Sperm cells at different stage of development. Scale bars: **A** 200 μm; **B–N** 50 μm.

**Figure 7. F7:**
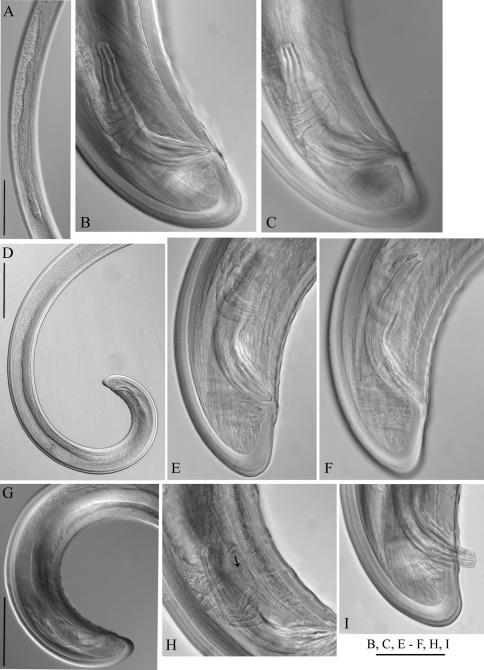
*Longidorus carniolensis* sp. n. *Male:* **A** Posterior genital branch **B, C, E, F** Tail and copulatory apparatus – different optical sections **D, G** Posterior end **H** Rectum (marked by arrow), spicules and lateral piece **I** Partly protracted spicules. Scale bars: **A, D, G** – 200 μm; **B, C, E–F, H, I** – 50 μm.

**Figure 8. F8:**
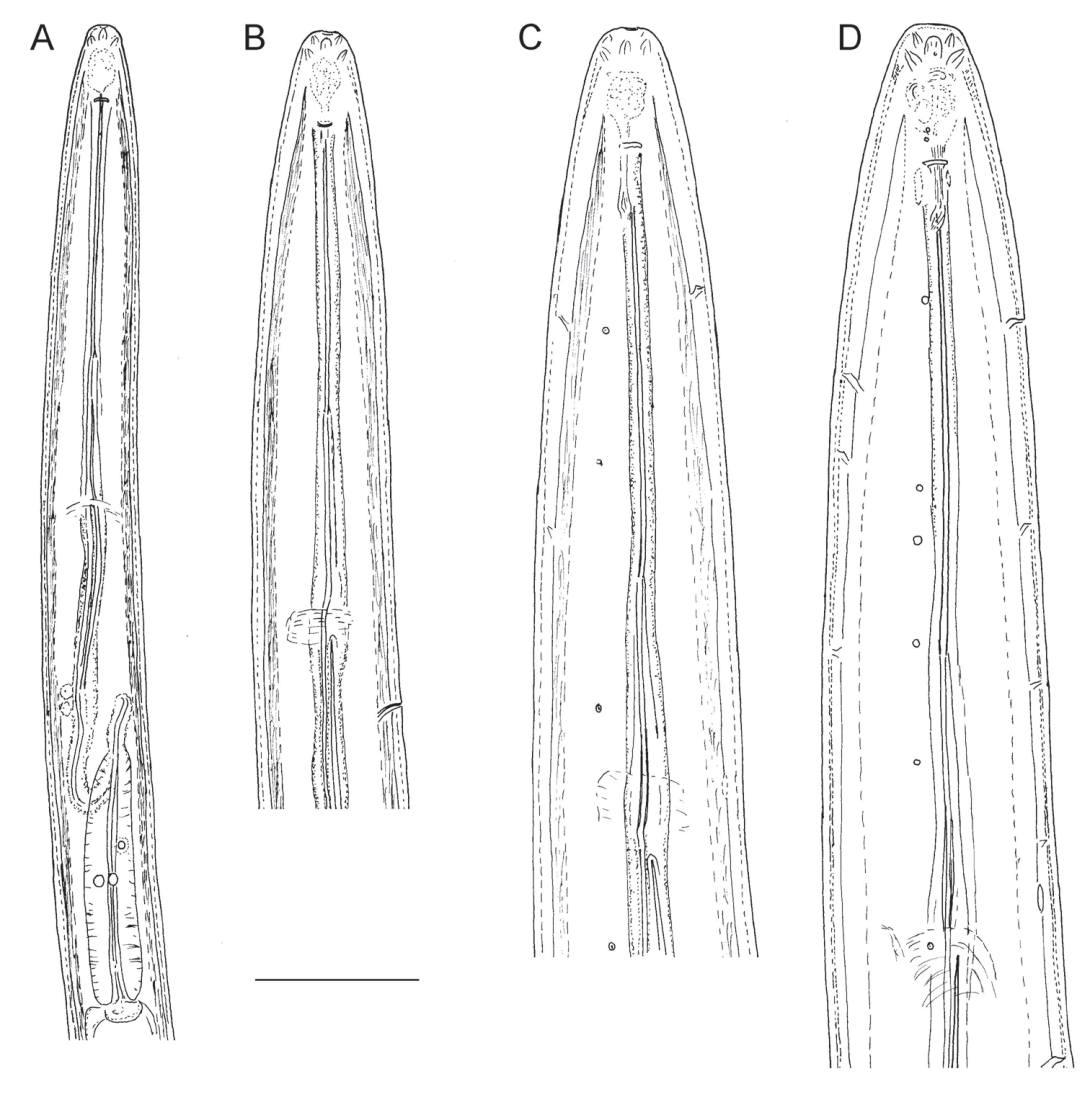
*Longidorus carniolensis* sp. n. *Juveniles*: **A** Neck region of first stage **B–D** Spear region of second, third and fourth stage. *Scale bar*: 50 μm.

**Figure 9. F9:**
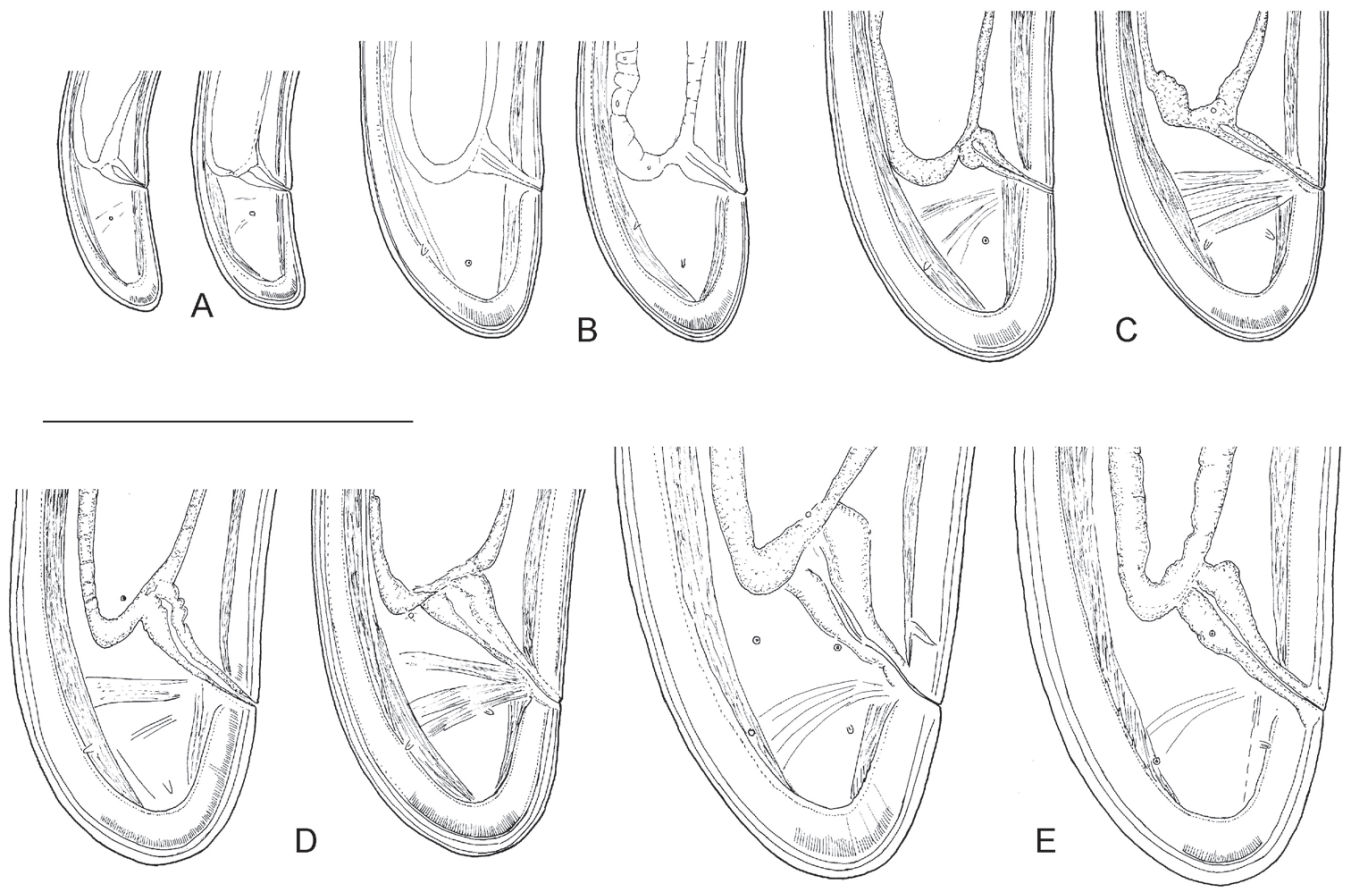
*Longidorus carniolensis* sp. n. Evolution of the tail. **A–D** Tail of first–fourth juvenile stage **E** Tail of female. Scale bar: 100 μm.

**Figure 10. F10:**
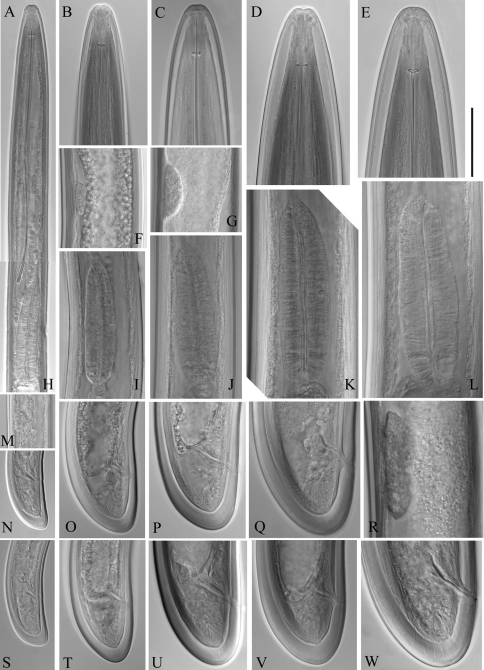
*Longidorus carniolensis* sp. n. *Juvenile*: **A–D** Anterior region of first, second, third and forth stages **H–K** Pharyngeal bulb of first, second, third and forth juvenile stages **M, F, G, R** genital primordium of first, second, third and forth stages **N, S** Tail shape of first stage **O, T** Tail shape of second stage **P, U** Tail shape of third stage **Q, V** Tail shape of forth stage *Female*: **E** Anterior region **L** Pharyngeal bulb **W** Tail shape. *Scale bar:* 50 μm.

**Figure 11. F11:**
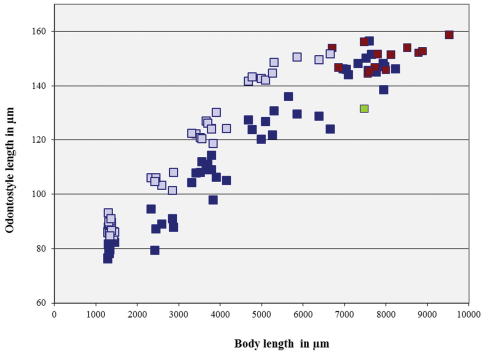
*Longidorus carniolensis* sp. n. Scatter plot of the functional (dark blue) and replacement odontostyle (light blue) in relation to the body length of the juvenile stages and adults: females (dark blue) and males (red), female with very short odontostyle (green).

#### Differential diagnosis and relationships. 

*Longidorus carniolensis*is an amphimictic species**,** characterized by females with a moderately long (L=5.6–8.2 mm) and plump (**a**=51–72) body, assuming a spiral to C-shape when heat relaxed; head region continuous, anteriorly almost flat, lip region 23–25 µm wide, guiding ring situated posteriorly (42–47 μm, 43–50 μm in males), long odontostyle (146.6 (136–157) μm), distribution of pharyngeal glands normal, nuclei of approximately equal size, tail bluntly conoid to hemispherical. Males abundant, spicules slender and long (122–145 μm), ventromedian supplements 13–17, irregularly spaced and preceded by an adanal pair. Postembrional development through four juvenile stages.

The codes for identifying the new species when using the polytomous key by Chen et al. (1997) are: A 56, B 4, C 4, D 1, E 4, F 35, G 1, H 1, I 2. The species belongs to the group of species with long odontostyle – over 100 μm and bluntly conoid to hemispherical tail: *Longidorus poessneckensis*, *Longidorus macrosoma*, *Longidorus caespiticola*, *Longidorus helveticus*, *Longidorus macroteromucronatus* Altherr, 1974, *Longidorus raskii* Lamberti & Agostinelli, 1993, *Longidorus kheirii* Pedram, Niknam, Robbins, Ye & Karegar, 2008, *Longidorus pius* Barsi & Lamberti, 2001*, L. nevesi* Macara, 1986, *Longidorus major* Roca & d’Erico, 1987, *Longidorus carpathicus*, *Longidorus piceicola*, *Longidorus vinearum* Bravo & Roca, 1995, *Longidorus pauli* Lamberti, Molinari,De Luca, Agostinelli & Di Vito, 1999, *Longidorus arthensis*, *Longidorus iuglandis* Roca, Lamberti & Agostinelli, 1985, *Longidorus picenus* Roca, Lamberti & Agostinelli, 1985, *Longidorus silvae* Roca, 1993, *Longidorus uroshis*, *Longidorus saginus* Khan, Seshardi, Weischer & Mathen, 1971, *Longidorus orongorongensis* Yeates & Van Etteger, 1992, *Longidorus cretensis* Tzortzakakis, Peneva, Terzakis, Neilson & Brown, 2001, *Longidorus cylindricaudatus* Krnjaić, Roca, Krnjaic & Agostinelli, 2005, *Longidorus fasciatus* Roca & Lamberti, 1981 and *Longidorus litchii*. *Longidorus carniolensis* sp. n. can be differentiated from all these species either by morphometrics or/and quantitative characters. It differs from:

*Longidorus poessneckensis* – by its somewhat longer odontostyle (ave. 147.5 (136–157) *vs* ave. 133 (122–142), ave. 126 (122–130) and ave. 140.2 (132–148) μm); more posteriorly sitated guiding ring (ave. 44.6 (42–47) *vs* ave. 40 (36–43) and 39 ave. (37–40) μm); tail short conoidal *vs* elongate conoid in J1 (c’=1.2–1.5 *vs* 1.8–2.2 and 1.8–2.5); males abundant *vs* males very rare (Sturhan and Loof 2001, Lišková and Kumari 2010, Kornobis and Peneva 2011);

*Longidorus macrosoma* – by its shorter (5.6–8.2 *vs* 8.4–11.9 mm) and more plump (a=51–72.4 *vs* 77–113 and 105–126) body; differently shaped lip region (laterally rounded *vs* truncated, flattened); somewhat longer odontostyle (136–157 *vs* 113–148 μm); different tail shape in J1, bluntly conoidal *vs* mucronate; longer spicules in males 122–145 *vs* 105 μm and ave. 116.2 (112–121) μm (Hooper 1961, Lamberti et al. 2001);

*Longidorus caespiticola* – by its longer odontostyle (136–157 *vs* 100–120, 96–109 μm); different numbers of dorsal (2–5 *vs* a single pore) and ventral (7–10 *vs* 5–6) pores; more posteriorly sitated guiding ring (42–47 *vs* 30–41, 37, 42.5 μm); longer spicules in males (122–145 *vs* 90, 80–95 μm), smaller **c’** value in J1 (1.2–1.5 *vs* almost 2) (Hooper 1961, Širca and Urek 2009);

*Longidorus helveticus* – by different tail shape of J1 being bluntly conoid *vs* mucronated; longer (122–145 *vs* 104–118 μm ) and differently shaped spicules (Lamberti et al. 2001);

*Longidorus macroteromucronatus* – by having more posteriorly situated guide ring (42–47 vs 38 μm); thicker cuticle along the body (6–7 *vs* 4 μm) and on tail region (9–10.5 v*s* 13–15 μm); longer odontostyle (136–157 *vs* 133 μm) (Altherr 1974);

*Longidorus raskii* – by its wider lip region (23–25 *vs* 15–19 and 14–16 μm); more posteriorly situated guiding ring (42–47 *vs* 33–38 and 33–43 μm); longer odontostyle (136–157 *vs* 90–103 and 98–100 μm);longer spicules (122–145 *vs* 82–103 and 79–90 μm) (Lamberti and Agostinelli 1993, Lamberti et al. 2001, Krnjaić et al. 2002);

*Longidorus kheirii* – by itslonger odontostyle (136–157 *vs* 111–130 μm); different tail shape in J1 (bluntly conoidal *vs* mucronated); males abundant *vs* males rare; longer spicules (122–145 *vs* 80 μm) (Pedram et al. 2008);

*Longidorus pius* – by its more posterior position of the guiding ring (42–47 *vs* 35–42 and 37–42.5 μm); different tail shape in J1 (bluntly conoidal *vs* mucronated); males abundant *vs* males absent (Barsi and Lamberti 2001a, Barsi and De Luca 2008)

*Longidorus nevesi* – by having wider lip region (23–25 *vs* 16–22 μm), different amphidial fovea shape (pouch like, not bilobed *vs* bilobed); differently shaped and longer spicules in males (122–145 *vs* 87–100 μm) (Macara 1986);

*Longidorus major* – by having shorter body (L=5.6–8.2 *vs* 8.5–12 mm); somewhat narrower lip region (23–25 *vs* 22–27 μm); different tail shape in J1 (bluntly conoidal *vs* mucronate) and amphidial fovea (pouch like, not bilobed *vs* bilobed), males abundant *vs* males absent (Roca and d’Erico 1987);

*Longidorus carpathicus* – by its longer body (L=5.6–8.2 mm *vs* 6.2–6.5 mm); wider (23–25 *vs* 16–18 μm) and differently shaped lip region; lower **c’** value (**c’**= ave. 0.6 (0.5–1.0) *vs* **c’**= 0.8); different shape in J1 (bluntly conoidal *vs* mucronated with a rather long mucro); males abundant *vs* males absent (Lišková et al. 1997);

*Longidorus piceicola* – by having longer body (L=5.6–8.2 *vs* 4.2–6.5, 4.4–8.0 and 5.2–7.9 mm); wider (23–25 *vs* 14–17 μm) and differently shaped lip region (continious, almost flat *vs* broadly rounded); lower **c’** value (**c’**=ave. 0.6 (0.5–1.0) *vs* **c’**=0.9–1.3); differently shaped tail in J1 (bluntly conoidal *vs* elongate conoid) (Lišková et al. 1997, Barsi and Lamberti 2001b);

*Longidorus vinearum* – by having different lip region shape (abruptly *vs* gradually tapering), different shape of amphidial fovea (pouch like not bilobed *vs* irregularly bilobed); longer odontostyle (136–157 *vs* 105.5–132.5 μm); different tail shape in J1 (bluntly conoidal *vs* conical, c’=1.2–1.5 *vs* 1.9–2.8) (Bravo and Roca 1995);

*Longidorus pauli* – by having different (continious *vs* slightly offset) and wider (23–25 *vs* 14–17 μm) lip region, amphidial fovea pouch like, not bilobed *vs* bilobed; longer odontostyle (136–157 *vs* 102–118 μm); lower **a** and **c’** values (**a**=51.0–72.4 *vs* **a**=120.3–143.5; **c’**= ave. 0.6 (0.5–1.0) *vs* **c’**=0.9 (0.8–1.0), respectively); more posteriorly situated guiding ring (42–47 *vs* 27–36 μm); longer spicules (122–145 *vs* 61–69 μm); different tail shape in J1 (bluntly conoidal *vs* subdigitate) (Lamberti et al. 1999);

*Longidorus arthensis* – by itswider (23–25 *vs* 14–17 μm) lip region, amphidial fovea pouch like not bilobed *vs* bilobed; longer odontostyle (136–157 *vs* 102–111 μm); lower **c’** values (**c’**=av 0.6 (0.5–0.1) *vs* **c’**=av 0.9 (0.8–1.1); more posteriorly sitated guiding ring (42–47 *vs* 30–38 μm); longer spicules (122–145 *vs* 60–66 μm); different tail shape in J1 (bluntly conoidal *vs* mucronated) (Brown et al. 1994);

*Longidorus iuglandis* – by its wider lip region (23–25 *vs* 14–16 μm); amphidial pouches not bilobed *vs* bilobed; longer odonostyle (136–157 *vs* 112–128 μm); more posterior position of the guiding ring (42–47 *vs* 31–41 μm); longer tail (34–69 *vs* 33–41 μm); longer spicules (122–145 *vs* 93–99 μm); different tail shape in J1 (bluntly conoidal *vs* mucronated) (Roca et al. 1985);

*Longidorus picenus* - by its wider lip region (23–25 *vs* 19–22 μm); amphidial fovea not bilobed *vs* bilobed; more posterior position of the guiding ring (42–47 *vs* 31–41 μm); longer spicules (122–145 *vs* 103–112 μm); different tail shape in J1 (bluntly conoidal *vs* mucronated) (Roca et al. 1985);

*Longidorus silvae* - by its more plump body (**a**=51.0–72.4 *vs* **a**=87.5–137.5 in Italian population and **a**=87.4–116 in Serbian populations), wider lip region (23–25 *vs* 14–17 μm); amphidial fovea not bilobed *vs* bilobed; longer odontostyle (136–157 *vs* 113.5–133 μm (Italian population) and 108–136 μm (Serbian populations)); different tail shape in J1 (bluntly conoidal *vs* mucronated); males abundant *vs* males rare; longer spicules (122–145 *vs* 77–78 μm) (Roca 1993, Barsi and Lamberti 2004, Barsi et al. 2007);

*Longidorus uroshis* – by having wider (23–25 *vs* 15–20.5 μm) lip region; lower **a** values (**a**=51.0–72.4 *vs* a=96.9–108.9); different tail shape in J1 (bluntly conoidal *vs* mucronated); longer spicules (122–145 *vs* 59–72 μm) (Krnjaić et al. 2000);

*Longidorus saginus* – by having longer body (L=5.6–9.5 *vs* 4.8–6.4 mm); amphidial fovea pouch shaped not bilobed *vs* asymetrically bilobed; longer tail (34–69 *vs* 21–33 μm) (Khan et al. 1971);

*Longidorus orongorongensis* – by its more anterior position of the guiding ring (42 -47 *vs* 63–73 μm); shorter odontostyle (136–157 *vs* 152–166 μm); longer spicules (122–145 *vs* 84–87 μm) (Yeates et al. 1992);

*Longidorus cretensis* – by having normal *vs* abnormal location of pharyngeal glands; wider lip region (23–25 *vs* 17–21 μm); longer spicules (122–145 *vs* 71–91 μm); different tail shape in J1 (bluntly conoidal *vs* conoid pointed) (Tzortzakakis et al. 2001);

*Longidorus cylindricaudatus* – by having lip region abruptly *vs* gradually tapering; amphidial fovea not bilobed *vs* bilobed; shorter odontostyle (136–157 *vs* 164–178 μm); lower **a** values (**a**=51–72.4 *vs* **a**=94.4–113.4); males abundant *vs* males absent (Krnjaić et al. 2005);

*Longidorus fasciatus* – by its wider lip region (23–25 *vs* 12–14 μm); different amphidial poches (not bilobed *vs* asymmetrically bilobed); longer odontostyle (136–157 *vs* 102–119 μm; male abundant *vs* males absent (Roca and Lamberti 1981);

*Longidorus litchii* – by its somewhat shorter odontostyle (136–157 *vs* 138–171 mm); different amphidial poches (not bilobed *vs* bilobed); more anterior postion of the guiding ring (42–47 *vs* 82.5–96.5 μm); different tail shape in J1 (bluntly conoidal *vs* elongate conoid with long digitate tip, **c’**=1.2–1.5 *vs* **c’**=2.7–3.4); longer spicules (122–145 vs 68.5–71 μm) (Xu and Cheng 1992).

#### Type-locality and plant association.

An old vineyard with roots of several *Vitis vinifera* varieties close to Drašiči village in southern part of Slovenia (45°39'N; 15°23'E), 229 m above sea-level.

Other-locality: a vineyard close to Krmačina village in southern part of Slovenia.

#### Distribution notes.

*Longidorus carniolensis* n. sp were detected in 6 out of 10 soil samples from locations of Drašiči and Krmačina. Population density was 4–15 specimens of all developmental stages per 200 cm^3^ of soil sample.

#### Type-material.

Holotype female and 2 female, 5 male and 8 juvenile (3 J1, 1 J2, 2 J3, 2 J4) paratypes deposited in the Nematode Collection of Agricultural Institute of Slovenia, Ljubljana, Slovenia; two female, two male and 10 juvenile (3 J1, 2 J2, 2 J3, 2 J4) paratypes - in the Wageningen Nematode Collection (WaNeCo), Wageningen, the Netherlands; one female, one male and 6 juvenile (3 J1, 1 J2, 1 J3, 1 J4) paratypes - in the Nematode Collection of The Food and Environment Research Agency, Sand Hutton, UK (former Rothamsted Nematode Collection); one female, three male and 6 juvenile (3 J1, 2 J3, 1 J4) paratypes - in the Nematode Collection of the Zoology Museum, Ghent University, Belgium; one female, three male and two juvenile (1 J3, 1 J4) paratypes - in the Nematode Collection of the Institute of Plant Protection, Bari, Italy; two female, one male and 6 juvenile (3 J1, 1 J2, 1 J3, 1 J4) paratypes - in the Nematode Collection of the University of California at Riverside, USA; one female, one male and two juvenile (1 J3, 1 J4) paratypes - in the USDA Nematode Collection, Beltsville, Maryland, USA; 4 female, 5 male and 8 juvenile (3 J1, 1 J2, 2 J3, 2 J4) paratypes - in the Nematode Collection of the Institute of Biodiversity and Ecosystem Research, BAS, Sofia.

#### Etymology.

The species epithet *carniolensis* was derived from Carniola which is the Latin name of the Kranjska province, a historical region that comprised parts of what is now Slovenia.

#### rDNA sequence analysis.

**Figure 12.**
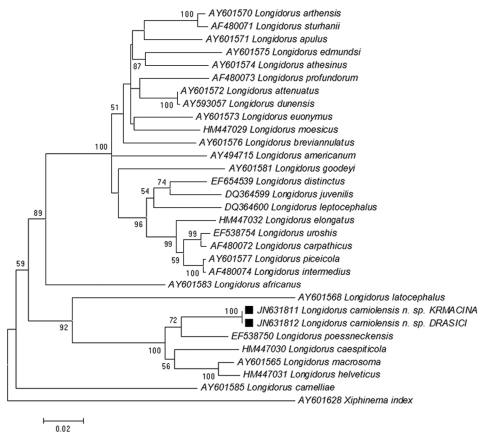
Phylogenetic tree of rDNA D2/D3 expansion region sequences of *Longidorus carniolensis* sp. n. from Slovenia (square mark) and sequences of closely related *Longidorus* species (NCBI GenBank). Sequences were analysed using Neighbour Joining Method. Bootstrap support values higher than 50% are presented.

Cluster analyses of the D2-D3 expansion regions of the 28S rDNA nuclear gene sequences of *Longidorus carniolensis* sp. n. and closely related species (Table 1) were performed and a phylogenetic tree was constructed (Fig. 12). The sequences of both populations of *Longidorus carniolensis* sp. n.from Drašiči and Krmačina were identical. They formed a distinct clade within a cluster of the closely related sequences of *Longidorus poessneckensis*, *Longidorus helveticus*, *Longidorus macrosoma*, *Longidorus caespiticola* and *Longidorus latocephalus*. The closest sequence to *Longidorus carniolensis* sp. n.was the sequence of *Longidorus poessneckensis* (Acc. No EF538750) with 91.9% of similarity.

## Discussion

There are some characteristic morphological features observed in *Longidorus carniolensis* sp. n. such as the presence of vestigium, hemizonid and hemizonion, and the abberant ventral pore. The vestigium was present in all specimens (males and females), it was located in the slender pharynx, behind odontophore, in few specimens in the odontophore area. Such a vestigium has been reported also for *Longidorus fursti* Heyns, Coomans, Hutsebaut et Swart, 1987 from South Africa; two Chinese *Longidorus* species (Xu and Cheng 1992); it is more frequently observed in *Xiphinema* spp. (Kruger and Heyns 1987, Swart 1994, Swart and Quénéhervé 1998; Mincheva et al. 2008), reported also for several species of *Xiphidorus* (Decraemer et al. 1996) and *Paraxiphidorus brevistylus* (Decraemer et al. 1998).

Hemizonid and hemizonion are not commonly observed structures in dorylaimids (Jairajpuri and Ahmad 1992), the hemizonid was seen both in adults and in the last two juvenile stages of the new species and hemizonion in only two male specimens. Both structures were reported also for *Longidorus fursti* (Heyns et al. 1987), *Longidorus iranicus* (Sturhan and Barooti 1983); only hemizonid – for *Longidorus litchii*, *Longidorus henanus* (Xu and Cheng 1992). *Longidorus carpetanensis* Arias, Fé Andres & Navas, 1986, *Longidorus pawneensis* Luc & Coomans, 1988, *Longidorus brevis* Swart et al., 1996, *Longidorus africanus* (Bravo and Roca 1995), *Longidorus kheirii* (Pedram et al. 2008), *Longidorus laevicapitatus* Williams, 1959 (Heyns and Luc 1987), in a few specimens of *Longidorus fagi* Peneva, Choleva & Nedelchev, 1997, for one *Xiphidorus* and some *Paralongidorus* species (Siddiqi et al. 1963, Fisher 1964, Luc and Doucet 1984).

The only available data on the excretory system in *Longidorus* refers to *Longidorus macrosoma* in which a ventral excretory pore at the level of the nerve ring, leading to a non-canalicular tissue in its anterior part has been observed together with two nucleated glands embedded in the tissues of a ventrally located ampulla-like structure (Aboul-Eid 1969). In *Longidorus carniolensis* sp. n. we observed an aberrant ventral pore in all adults, differing in structure from the other ventral pores and also having a longer duct (Figs 1A, 4F and 6H), it probably functions as more specialised part of the excretory system. It was also detected in juvenile stages (Fig. 8B, D).

The data on D2D3 rDNA regions of majority of longidorid species, particularly of those belonging to the genus *Longidorus* is far to be complete; this does not facilitate the reconstruction of the phylogenetic relationships among the members of this widely distributed group of ectoparasitic nematodes. Despite of this, based on the rDNA results as well as a combination of morphological features the new species is included in a clearly defined group of closely related species (*Longidorus poessneckensis* (92% similarity), *Longidorus macrosoma* and *Longidorus caespiticola* (90%), *Longidorus helveticus* (89%), sharing some common characters – amphids with pouch-like fovea, not bilobed, amphidial duct well discernable, tapering lip region, which is continuous with the rest of body, normal arrangement of pharyngeal glands, bluntly conoidal to hemispherical tail, much shorter or equal to the anal body width; and the development through 4 juvenile stages. All these species occur in Europe, more frequently in West and Central Europe. The correlation between the amphid structure and clustering of longidorid species has been underlined by Rubtsova et al. (2001) and He et al. (2005) and it is supported by our study.

## Supplementary Material

XML Treatment for Longidorus carniolensis
